# Surgical treatment for diffused-type giant cell tumor (pigmented villonodular synovitis) about the ankle joint

**DOI:** 10.1186/s12891-017-1824-6

**Published:** 2017-11-14

**Authors:** Xingchen Li, Yang Xu, Yuan Zhu, Xiangyang Xu

**Affiliations:** Orthopaedic Department, Ruijin Hospital, Ruijin Er Road No.197, Shanghai, 200025 China

**Keywords:** Giant cell tumors, Ankle joint, Joint preserving surgery, Bone transplantation

## Abstract

**Background:**

Diffused-type giant cell tumor(Dt-GCT) is a rare, aggressive disorder of the joint synovium, bursa and tendon sheaths. Osseous erosions and subchondral cysts may develop as the result of synovium infiltration in Dt-GCT. We present a retrospective study of a series of patients who are diagnosed with Dt-GCT about the ankle joint, there clinical outcome is evaluated in this study.

**Material and method:**

Fifteen patients with radiologically and histologically confirmed Dt-GCT about the ankle joint were identified in our foot and ankle department. Patients were managed with open synovectomy for the tumor tissue and bone grafting for bony erosions. X-rays and MRI scans were used for evaluation of the tumor and bony erosions pre- and post-operatively. Pre- and post-operative ankle function was assessed using the American Orthopedic Foot and Ankle Society –Ankle and Hindfoot (AOFAS-AH) score and the Muscularskeletal Tumor Society (MSTS) score.

**Results:**

The mean follow-up duration was 37.4 months (range 25 to 50 months). There were 6 males and 9 females, with a mean age of 35 years old (range 18 to 65 years). All patients had talar erosion with the average size of 10.1*9.1*8.2 mm, distal tibia was affected in 5 patients with the average size of 6.2*5.6*5.8 mm. 7 patients had tendon involvement, 2 patients had recurrence and progression of ankle osteoarthritis. Both of them underwent ankle fusion. At the time of last follow-up, the mean AOFAS-AH score increased from 49 to 80 points (*p* < 0.05), the MSTS score increased from 12 to 22 points (p < 0.05).

**Conclusion:**

For Dt-GCT with bony erosions, open synovectomy combined with bone grafting seems to be a safe and effective operation for the salvage of ankle joint. Fusion is recommended for failed and severe cartilage destruction of the ankle joint.

## Background

Giant cell-rich tumors(GCT) that arise from tendons and synovium are now classified into two forms: localized and diffuse. In the World Health Organization(WHO) classification, the former is described as giant cell tumor of tendon sheath(GCT-TS), whereas the later is described as diffused-type giant cell tumor(Dt-GCT), also known as pigmented villonodular synovitis(PVNS) [1, 2].

GCT-TS is characterized by focal involvement of the synovium, tendon sheath or bursa, with nodular or pedunculated masses. While the Dt-GCT is relatively rare, benign and locally aggressive [3]. It is featured with the osseous erosions and subchondral cysts resulted from synovial infiltration [2, 4–7]. The etiology for Dt-GCT is still controversial and has not been well established in literature. Chronic inflammatory disease [8, 9] or a history of trauma [10] may cause Dt-GCT.

The most common joint involvement of Dt-GCT include knee, hand joints and hip, followed by ankle and shoulder [10–12]. Dt-GCT about the ankle joint accounts for approximately 2.5% of the cases [11]. Patients may present with insidious pain, swollen and stiffness of the affected ankle joint which may has presented for months or years. Physical examination can find swollen and restricted range of motion of the ankle joint. Subtalar joint or even mid-foot joints can also be affected in several severe diffused cases.

Subchondral cysts and osseous erosions can present in Dt-GCT about the ankle joint. Loss of bony structure and volume is typical at sites of fracture and fusion. We hypothesis that for mild to moderate bony erosion of the ankle joint, open synovectomy combined with impaction bone grafting can be able to reduce symptoms and prevent further destruction of the talar cartilage and progression of the ankle osteoarthritis.

The aim of this study is to report our experience in the management of Dt-GCT about the ankle joint, and also to evaluate the clinical outcome of open synovectomy combined with bone grafting.

## Methods

**Fig. 1:**
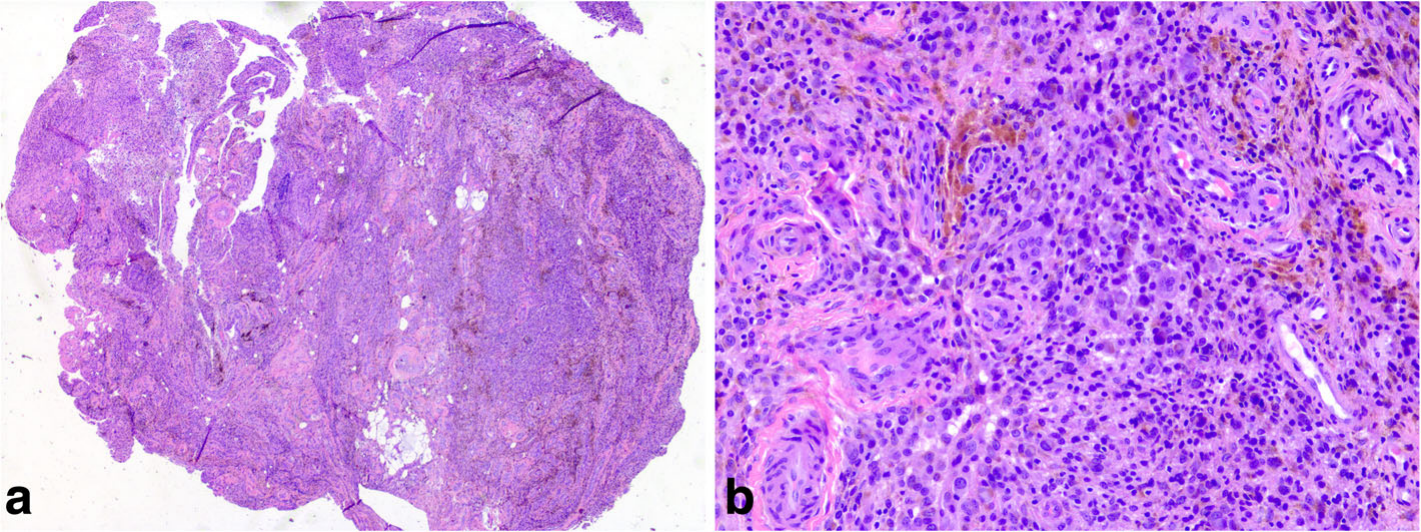
The photomicrographs demonstrate the mixture of multinucleated giant cells, mononuclear cells, foam cells and hemosiderin deposits **a**, Low-power view (H&E stain, original magnification ×25); **b**, High-power view (H&E stain, original magnification ×200)

We retrospectively reviewed a total of 15 patients with radiologically and histologically confirmed Dt-GCT about the ankle joint (Fig. 1). Patient demographics include age, sex, side, symptom and follow-up duration, size of bony erosions, patient satisfaction were recorded (Table 1).

**Table 1:** Patient demographics

NO.	FU (M)	Side	Symptom Duration (M)	Bony Erosions (mm)	Talar Lesion Management	Additional Surgery	Patient Satisfaction
1	38	R	18	Talus: 10*13*8, 12*10*9, Distal Tibia: 5*5*7	OATS (Ø10mm, 2 plugs)	–	Good
2	26	R	24	Talus: 10*9*7, Distal Tibia: 11*8*10, Distal fibular: 8*12*8	Bone grafting (allogenic cancellous bone)	Modified Brostrom	Good
3	27	L	15	Talus: 7*6*6	Bone grafting (allogenic cancellous bone)	Modified Brostrom	Fair
4	43	R	12	Talus: 10*10*8, Distal Tibia: 4*4*3	OATS (Ø10mm,2 plugs)	Syndesmosis screw fixaion	Fair
5	25	L	10	Talus: 6*5*7	Bone grafting (allogenic cancellous bone)	Modified Brostrom	Excellent
6	44	R	16	Talus: 9*6*8	Bone grafting (allogenic cancellous bone)	Modified Brostrom	Good
7	30	R	15	Talus: 7*5*5	Bone grafting (allogenic cancellous bone)	–	Good
8	45	L	12	Talus: 5*9*7, Distal Tibia: 5*6*5	Bone grafting (allogenic cancellous bone)	–	Fair
9	37	L	10	Talus: 8*8*5	Bone grafting (allogenic cancellous bone)	Modified Brostrom	Bad
10	50	R	6	Talus: 14*9*11	OATS (Ø10mm, 2 plugs)	–	Bad
11	42	R	30	Talus: 6*7*8, Distal Tibia: 6*5*4	Bone grafting (allogenic cancellous bone)	–	Excellent
12	31	R	24	Talus: 10*11*9, Subtalar joint	Bone grafting (allogenic cancellous bone)	Modified Brostrom	Fair
13	49	L	12	Talus: 14*9*10	OATS (Ø10mm, 2 plugs)	–	Excellent
14	34	R	18	Talus: 9*8*8	Bone grafting (allogenic cancellous bone)	Modified Brostrom	Good
15	40	L	15	Talus: 14*12*7	OATS (Ø10mm, 2 plugs)	–	Fair

The diagnosis of Dt-GCT was made according to patient history, clinical manifestation, MRI findings and typical histological features. MRI scans were obtained under suspicion of Dt-GCT. The location and extent of involvement of the tumor were further evaluated by MRI, as well as the adjacent soft tissue, joints and tendon sheaths involvement.

MRI scans were also helpful to plan the appropriate surgical approach [13]. The surgical approach was carefully designed preoperatively based on the location of tumor tissue and bony erosions. Patients were managed with open synovectomy for the tumor tissue and bone grafting for bony erosions. No radiotherapy was used for any patient.

**Fig. 2:**
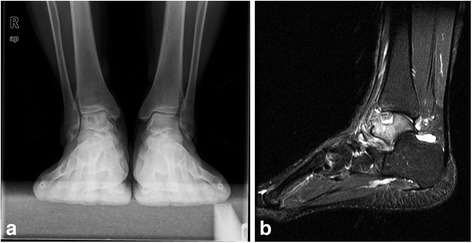
**a** Weight bearing Anterioposterior view of the ankle joint showed a lucent area at the talus; **b** A large subchondral cyst wad identified with high signal on SIRT image

**Fig. 3:**
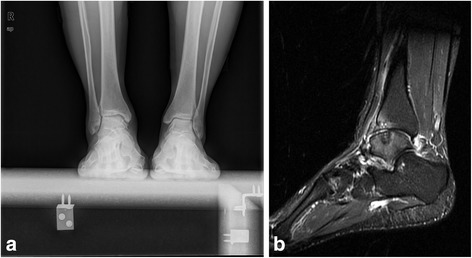
**a**, an anteriomedial incision was made for the exposure of the tumor tissue and talus; **b**, two osteochondral autografts were used to fill the talar defects

**Fig. 4:**
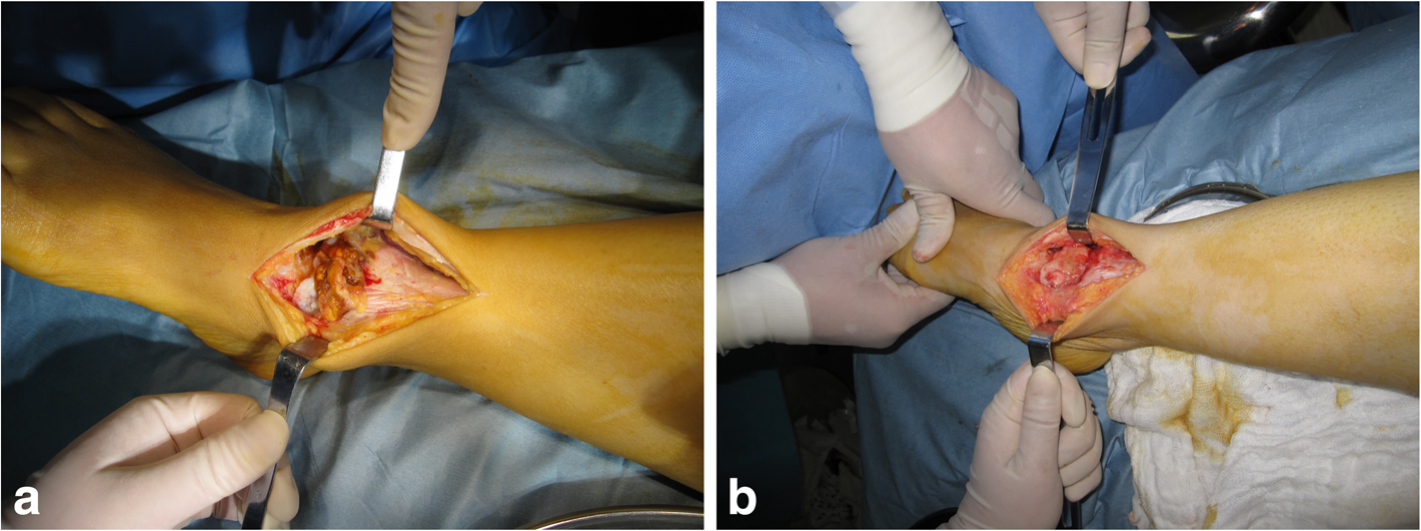
**a**, Weight bearing Anterioposterior view of the ankle joint showed improvement of the subchondral cyst in talus at 31 months after surgery; **b**, The SIRT image displayed the healing of the autograft to the surrounding tissue, the improvement of the bone marrow edema

Open synovectomy was performed for the treatment of Dt-GCT about the ankle joint. Based on the location of the tumor tissue and bony erosions, anterior or medial and lateral two incision approaches were used for exposure of the ankle joint. Bony erosions were identified and carefully debrided at the surgery, followed by impaction bone grafting to prevent the further fracture or collapse of the talar cartilage. Allogenic cancellous bone (Osteorad Ltd., Shanxi, China) was used for impaction bone grafting, while osteochondral autograft transplantation was considered when localized cartilage defect (greater than 10 mm in diameter) was identified at the surgery. Special instruments (osteochondral autograft transfer system, Arthrex, USA) were used for osteochondral autograft transplantation. Cylindrical autologous osteochondral plug, 8 or 10 mm in diameter taken from the ipsilateral knee joint was used for reconstruction of the talar defect. The medial upper part of the femoral chondyle was preferred as the donor site (Figs. 2, 3 and 4).

Functional outcome was evaluated using the American Orthopedic Foot and Ankle Society –Ankle and Hindfoot (AOFAS-AH) score and the Muscularskeletal Tumor Society (MSTS) score before and after surgery. The MSTS score was based on three general factors (pain, function, emotional acceptance) and three lower limb factors (use of supports, ability to walk and gait) [14]. Each category had a maximum score of 5 points and the total score was 30 points. The AOFAS-AH score was subdivided into pain (maximum 40 points), function (maximum 50 points), and alignment (maximum 10 points). Patient satisfaction level was graded as excellent, good, fair or poor.

SAS software (version 8.02, SAS Institute, USA) was used for statistical analysis. Paired student t-test was conducted for the evaluation of improvement in AOFAS-AH score and MSTS score. A *P* value less than 0.05 was considered to be statistically significant.

## Results

The mean follow-up duration was 37.4 months (range 25 to 50 months). There were 6 males and 9 females in this study, with a mean age of 35 years old (range 18 to 65 years) at the time of surgery. The right ankle was involved in 9 patients (60%) and the remaining 6 patients (40%) had their left ankle got involved. 12 patients (80%) had a history of ankle trauma, while the remaining 3 patients (20%) denied any history of trauma. On average, symptoms presented for 15.8 months (range, 6 to 30 months) before the patient sought operative intervention. MRI scans were obtained pre-operatively for further evaluation of the tumor and surrounding tissue infiltration. The posterior tibialis tendon was involved in 4 patients (26.7%), the flexor hallux longus tendon was involved in 2 patients (13.4%), the flexor digitorum longus tendon was involved in 1 patient (6.7%) and the peroneal tendons were involved in 2 patients (13.4%). The subtalar joint was involved in 1 patient (6.7%) and the syndesmosis was involved in 1 patient (6.7%). The mean size of talar erosion was 10.1*9.1*8.2 mm, the distal tibia was involved in 5 patients (33.3%) with the mean size of 6.2*5.6*5.8 mm.

Open synovectomy was performed for all patients. Anterior approach was preferred in 7 patients (46.7%), medial and lateral two incisions were used in 8 patients (53.3%). For bony erosions, allogenic cancellous bone grafting was performed in 10 patients (66.7%), and the remaining 5 patients (33.3%) were managed with osteochondral autograft transplantation. No donor site morbidity was reported at the time of last follow-up.

2 patients with large bony erosions refused to receive ankle fusion as a primary surgery. One of them was 35 years old and the other was 48 years old, both of them had a strong desire to preserve their ankle joints. So open synovectomy, debridement of the subchondral cysts and bone grafting were performed for salvage of the ankle joint. Though mild stiffness and pain of the ankle joint was noticed, both of them were satisfied with the surgery at the time of last follow-up,

1 patient underwent syndesmosis screw fixation as the result of bony erosion at the site of distal tibiofibular syndesmosis. In 7 patients (46.7%), the lateral ligament was thought to be inadequate to restore the stability of the ankle joint as the result of extensive open synovectomy, the additional modified Brostrom procedure were performed to restore the stability of the ankle joint.

**Fig. 5:**
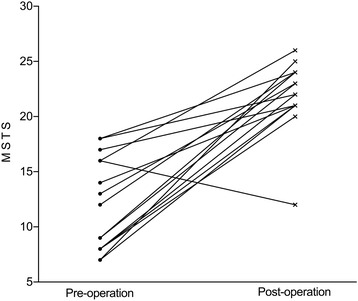
MSTS score compared pre- and postoperatively

**Fig. 6:**
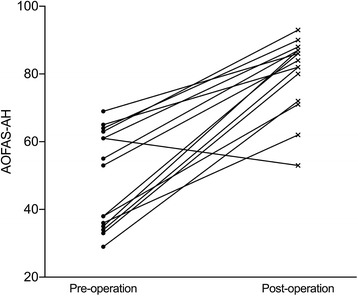
AOFAS-AH score compared pre- and postoperatively

The Muscularskeletal Tumor Society (MSTS) score and the AOFAS-AH questionnaires were used to assess the functional outcome of the surgery. The mean MSTS score increased from 12 pre-operatively to 22 points post-operatively at the time of last follow-up (*t* = 6.8, *p* < 0.05) (Fig. 5). The mean AOFAS-AH score increased from 49 pre-operatively to 80 points post-operatively (*t* = 7.8, p < 0.05) (Fig. 6). The AOFAS-AH pain subscale improved from 11 pre-operatively to 30 points post-operatively. The function subscale improved from 28 pre-operatively to 40 points post-operatively, and the alignment subscale remained the same at 10 points.

In regard to the overall satisfaction of the operation at the time of last follow-up, 8 patients (53.3%) rated the results as excellent or good, 5 (33.3%) were fair, and the remaining 2 (13.3%) were bad.

Magnetic resonance imaging scans were available for 8 patients (53.3%) at the time of last follow-up. The MRI findings showed at least 70% defect fill in 5 patients. 6 patients (75%) had normal or nearly normal signal at repair sites, 5 patients (62.5%) had mild or no effusion and 4 patients (50%) showed mild or no underlying bone-marrow edema.

## Discussion

GCT involving tendon sheaths and synovium can be subdivided into two types: the localized and the diffuse [1, 2]. The diagnosis is achieved by clinical evaluation, radiological and histological examinations. Dt-GCT is characterized by the insidious infiltration of the synovial lining and leading to osseous erosions [2, 6]. For patients with Dt-GCT about the ankle joint, tendon sheaths, talus, distal tibia and subtalar joint can be affected. In review of the literature, researches reporting the management of Dt-GCT about the ankle joint are quite limited.

The etiology of bony erosion is still controversial, increased joint pressure or pannus infiltration may be the reason for bony invasion [15, 16]. Efforts have been made to identify more detailed pathologies of Dt-GCT and bony involvement, like expression of an osteoclast phenotype [17, 18] or colony- stimulating factor 1 [19], and production of matrix metalloproteinases (MMP) [20, 21].

Despite the totally different pathologies between osteochondral lesion of the talus and bony erosion in Dt-GCT, cyclic loading of the ankle joint may be one of the contributors to subchondral cysts in both entities [22, 23]. Once the subchondral bone plate is broken as the result of trauma or synovial infiltration, ankle fluid can be forced into the subchondral bone during cyclic loading of the ankle joint. Subchondral cysts could occur as the result of cyclic loading of the joint and stimulating nerve endings in the subchondral bone, causing symptoms [22, 23]. To our knowledge, open synovectomy without the management of subchondral cysts will not be able to adequately relieve pain [24].

Meanwhile, early diagnosis and management for Dt-GCT and bony erosions are also essential for preventing further bone and cartilage damage [2, 25–28]. Ankle joint sustains extremely high pressure during the stance phase, approximately 650 N/cm^2^, the pressure can be even much higher during running according the biomechanical tests [22, 29]. So loss of bony structure and volume is typical at the sites of fracture and fusion. Saxena A and his colleagues [25] reported their study of ten patients with Dt-GCT about the ankle joint, the mean follow-up duration was 4.5 years. 5 patients had bony involvement, they advocated that surgical management for these patients could include open synovectomy and bone grafting of the cystic areas, along with irrigation with hydrogen peroxide. In our study, all patients with bony erosions are also managed with bone grafting. The use of impaction bone grafting will reconstruct the bony defect and provide structural support to the surrounding talar cartilage. Meanwhile, bone grafting also helps to improve the osteoinductive and osteoconductive processes. It is osteoinductive, bringing growth factors, signaling molecules that will facilitate bone growth and also osteoconductive, providing a mineral and collagen scaffold for native cells [30, 31]. Thus, Simultaneous treatment for the tumor tissue and bony erosion is warranted [25].

However, there is some disputes over the management for bony erosions of Dt-GCT in literature. Stvenson et al. [2] presented 13 patients with Dt-GCT about the foot and ankle simply underwent open total synovectomy, 5 patients who had ankle joint erosions, the peri-articular erosions and cysts were curetted with no bone grafting. No radiotherapy was used after surgery. None of the 13 patients had recurrence and all of them achieved excellent ankle functions at final follow-up.

Recurrence is among the most common complications for GCT after operation. GCT-TS can be managed successfully with open total synovectomy. However, the recurrence rate can be high in Dt-GCT even after extensive excision of the tumor tissue. The reason for recurrence can be multifactorial, treatment strategies, surgeon’s experience, the severity of the primary lesion and the involvement of the bony structures. Though Dt-GCT is usually managed with open synovectomy, while arthroscopic synovectomy is usually indicated in localized GCT, no consensus about the treatment strategies has been reached. Most recently, Noailles T and his colleagues [32] reviewed the literature about open surgery or arthroscopic synovectomy for GCT-TS in 2017, 33 articles were selected in this review, involving 1448 individuals. They concluded that arthroscopic excision was effective for localized GCT-TS for all four joints (shoulder, hip, knee, ankle). While the efficacy of arthroscopic synovectomy had only been shown for the knee joint for Dt-GCT.

2 patients (Patient No.9 and Patient No. 10) (13.3%) had recurrence and progressed to severe ankle osteoarthritis after primary open synovectomy in this study. Both of them complained continues ankle swelling, restricted range of motion of the ankle joint after surgery. And even unable to walk due to severe ankle pain during ambulation. X-rays or MRI showed severe articular destruction and bony erosions about the ankle joint. Ankle fusion was recommended for both of them. No recurrence was found after ankle fusion. None of the patients had wound problems after operation. Though 4 patients still had mild to moderate pain about the ankle joint with restricted range of motion of the ankle joint, they were satisfied with the surgery. We did not find any evidence of recurrence or progression of osteoarthritis on X-rays or MRI. Oral medication and functional rehabilitation was recommended for these patients.

Radiation has been advocated for Dt-GCT in post-operative setting after incomplete resection or failed primary surgery [33–35]. Mollon B and his colleagues [36] reported a meta-analysis about the effect of surgical synovectomy and radiotherapy on the rate of recurrence of Dt-GCT in 2015, very low-quality evidence found that the rate of recurrence of Dt-GCT was reduced by peri-operative radiotherapy. And they suggested that open synovectomy or synovectomy combined with peri-operative radiotherapy for Dt-GCT is associated with a reduced rate of recurrence.

Limitations of our study include the retrospective nature, limited patient number and follow-up duration and the use of self-reporting scores. Further prospective studies and longer term studies on larger patient population will be needed to ultimately determine the efficacy of this technique.

## Conclusions

In conclusion, early diagnosis and management are essential for Dt-GCT about the ankle joint. Open synovectomy together with bone grafting seems to be a safe and effective operation for the salvage of the ankle joint. Fusion is recommended for failed and severe cartilage destruction of the ankle joint.

## Abbreviations

AOFAS-AH: American Orthopaedic Foot and Ankle Society-Ankle and Hindfoot
Dt-GCT: Diffused-type Giant Cell Tumor
GCT: Giant Cell-rich Tumors
GCT-TS: Giant Cell Tumor of Tendon Sheath
MSTS: the Muscularskeletal Tumor Society
PVNS: Pigmented Villonodular Synovitis
WHO: World Health Organization
